# 5-Bromo-3-cyclo­pentyl­sulfinyl-2,7-dimethyl-1-benzofuran

**DOI:** 10.1107/S1600536811056091

**Published:** 2012-01-14

**Authors:** Hong Dae Choi, Pil Ja Seo, Uk Lee

**Affiliations:** a Department of Chemistry, Dongeui University, San 24 Kaya-dong Busanjin-gu, Busan 614-714, Republic of Korea; b Department of Chemistry, Pukyong National University, 599-1 Daeyeon 3-dong, Nam-gu, Busan 608-737, Republic of Korea

## Abstract

In the title compound, C_15_H_17_BrO_2_S, the cyclo­pentyl ring adopts an envelope conformation. In the crystal, mol­ecules are linked by weak C—H⋯O hydrogen bonds. A slipped π–π inter­action occurs between the furan and benzene rings of adjacent mol­ecules [centroid–centroid distance = 3.892 (3) Å and slippage = 1.786 (3) Å]. The crystal structure also exhibits a weak C—Br⋯π [2.919 (3) Å] inter­action.

## Related literature

For the biological activity of benzofuran compounds, see: Aslam *et al.* (2009 ▶); Galal *et al.* (2009 ▶); Khan *et al.* (2005 ▶). For natural products with benzofuran rings, see: Akgul & Anil (2003 ▶); Soekamto *et al.* (2003 ▶). For the crystal structures of related compounds, see: Choi *et al.* (2011**a* ▶,b* ▶).
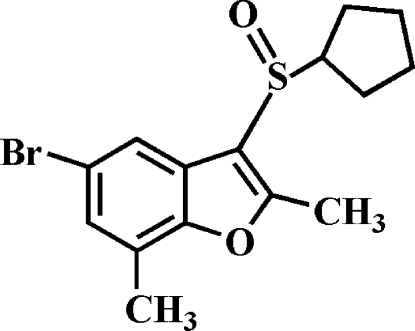


## Experimental

### 

#### Crystal data

C_15_H_17_BrO_2_S
*M* *_r_* = 341.26
Orthorhombic, 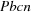

*a* = 19.5624 (8) Å
*b* = 8.3501 (4) Å
*c* = 17.5346 (7) Å
*V* = 2864.2 (2) Å^3^
*Z* = 8
Mo *K*α radiation
μ = 3.01 mm^−1^
*T* = 173 K
0.36 × 0.19 × 0.04 mm

#### Data collection

Bruker SMART APEXII CCD diffractometer
Absorption correction: multi-scan (*SADABS*; Bruker, 2009 ▶) *T*_min_ = 0.414, *T*_max_ = 0.899
14696 measured reflections
3561 independent reflections
2232 reflections with *I* > 2σ(*I*)
*R*_int_ = 0.052

#### Refinement

*R*[*F* ^2^ > 2σ(*F* ^2^)] = 0.043
*wR*(*F* ^2^) = 0.094
*S* = 1.01
3561 reflections
174 parameters
H-atom parameters constrained
Δρ_max_ = 0.43 e Å^−3^
Δρ_min_ = −0.51 e Å^−3^

### 

Data collection: *APEX2* (Bruker, 2009 ▶); cell refinement: *SAINT* (Bruker, 2009 ▶); data reduction: *SAINT*; program(s) used to solve structure: *SHELXS97* (Sheldrick, 2008 ▶); program(s) used to refine structure: *SHELXL97* (Sheldrick, 2008 ▶); molecular graphics: *ORTEP-3* (Farrugia, 1997 ▶) and *DIAMOND* (Brandenburg, 1998 ▶); software used to prepare material for publication: *SHELXL97*.

## Supplementary Material

Crystal structure: contains datablock(s) global, I. DOI: 10.1107/S1600536811056091/xu5434sup1.cif

Structure factors: contains datablock(s) I. DOI: 10.1107/S1600536811056091/xu5434Isup2.hkl

Supplementary material file. DOI: 10.1107/S1600536811056091/xu5434Isup3.cml

Additional supplementary materials:  crystallographic information; 3D view; checkCIF report

## Figures and Tables

**Table 1:** Hydrogen-bond geometry (Å, °)

*D*—H⋯*A*	*D*—H	H⋯*A*	*D*⋯*A*	*D*—H⋯*A*
C5—H5⋯O2^i^	0.95	2.60	3.465 (3)	152
C9—H9*B*⋯O2^i^	0.98	2.55	3.489 (3)	161
C10—H10*B*⋯O1^ii^	0.98	2.60	3.480 (3)	149

Symmetry codes: (i) 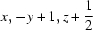; (ii) 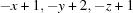.

**Fig. 1.**
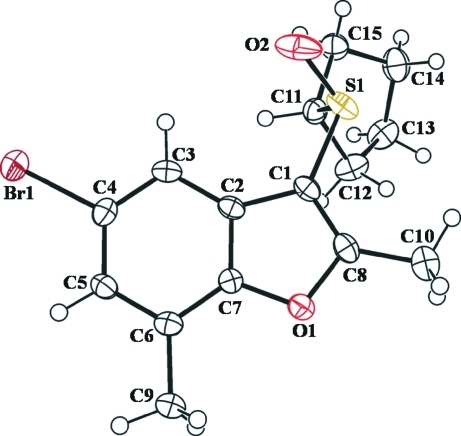
The molecular structure of the title compound with the atom numbering scheme. Displacement ellipsoids are drawn at the 50% probability level. H atoms are presented as small spheres of arbitrary radius.

**Fig. 2.**
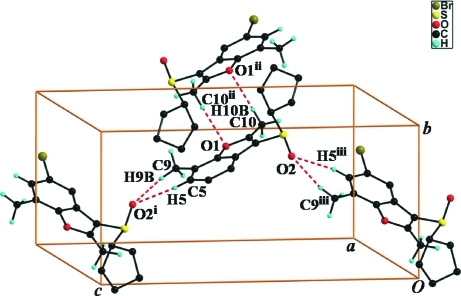
A view of the C—H···O hydrogen bonds (dotted lines) in the crystal structure of the title compound. H atoms non-participating in hydrogen-bonding were omitted for clarity. [Symmetry codes: (i) x, - y + 1, z + 1/2; (ii) - x + 1, - y + 2, - z + 1.]

**Fig. 3.**
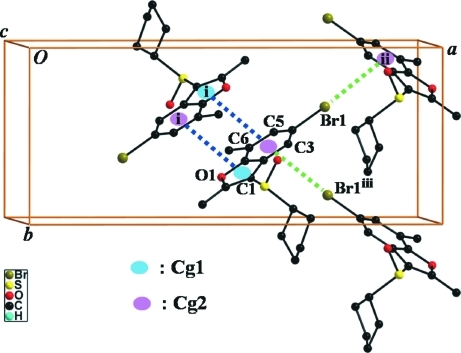
A view of the C—Br···π and π···π interactions (dotted lines) in the crystal structure of the title compound. All H atoms were omitted for clarity. [Symmetry codes: (i) 1 - x, 1- y, 1 - z; (ii) - x + 3/2, y - 1/2, z; (iii) - x + 3/2, y + 1/2, z. ]

## supplementary crystallographic information

### Comment

Benzofuran derivatives have drawn much interest in view of their valuable biological properties such as antibacterial and antifungal, antitumor and antiviral, and antimicrobial activities (Aslam *et al.*, 2009, Galal *et al.*, 2009, Khan *et al.*, 2005). These benzofuran derivatives occur in a wide range of natural products (Akgul & Anil, 2003; Soekamto *et al.*, 2003). As a part of our continuing study of 5-bromo-2,7-dimethyl-1-benzofuran derivatives containing either 3-cyclohexylsulfinyl (Choi *et al.*, 2011*a*) or 3-cyclohexylsulfonyl (Choi *et al.*, 2011*b*) substituents, we report herein the crystal structure of the title compound.

In the title molecule (Fig. 1), the benzofuran unit is essentially planar, with a mean deviation of 0.020 (1) Å from the least-squares plane defined by the nine constituent atoms. The cyclopentyl ring is in the envelope form. The crystal packing (Fig. 2) is stabilized by weak intermolecular C–H···O hydrogen bonds (see, Table 1). The crystal packing (Fig. 3) is further stabilized by a weak intermolecular C—Br···π interaction between the bromine and the benzene ring of an adjacent molecule, with a C4—Br1···Cg2^ii^ [2.919 (3) Å] (Cg2 is the centroid of the C2-C7 benzene ring). The crystal packing (Fig. 3) also exhibits a weak slipped π···π interaction between the furan and benzene rings of adjacent molecules, with a Cg1···Cg2^i^ distance of 3.892 (4) Å and an interplanar distance of 3.458 (3) Å resulting in a slippage of 1.786 (3) Å (Cg1 is the centroid of the C1/C2/C7/O1/C8 furan ring).

### Experimental

77% 3-chloroperoxybenzoic acid (202 mg, 0.9 mmol) was added in small portions to a stirred solution of 5-bromo-3-cyclopentylsulfanyl-2,7-dimethyl-1-benzofuran (260 mg, 0.8 mmol) in dichloromethane (30 mL) at 273 K. After being stirred at room temperature for 6 h, the mixture was washed with saturated sodium bicarbonate solution and the organic layer was separated, dried over magnesium sulfate, filtered and concentrated at reduced pressure. The residue was purified by column chromatography (hexane–ethyl acetate, 1:1 v/v) to afford the title compound as a colorless solid [yield 67%, m.p. 415–416 K; *R*_f_ = 0.51 (hexane–ethyl acetate, 1:1 v/v)]. Single crystals suitable for X-ray diffraction were prepared by slow evaporation of a solution of the title compound in benzene at room temperature.

### Refinement

All H atoms were positioned geometrically and refined using a riding model, with C—H = 0.95 Å for aryl, 1.00 Å for methine, 0.99 Å for methylene and 0.98 Å for methyl H atoms, respectively. *U*_iso_(H) =1.2*U*_eq_(C) for aryl, methine and methylene, and 1.5*U*_eq_(C) for methyl H atoms.

### Crystal data

**Table:**

C_15_H_17_BrO_2_S	*F*(000) = 1392
*M**_r_* = 341.26	*D*_x_ = 1.583 Mg m^−^^3^
Orthorhombic, *P**b**c**n*	Mo *K*α radiation, λ = 0.71073 Å
Hall symbol: -P 2n 2ab	Cell parameters from 2605 reflections
*a* = 19.5624 (8) Å	θ = 2.6–23.7°
*b* = 8.3501 (4) Å	µ = 3.01 mm^−^^1^
*c* = 17.5346 (7) Å	*T* = 173 K
*V* = 2864.2 (2) Å^3^	Block, colourless
*Z* = 8	0.36 × 0.19 × 0.04 mm

### Data collection

**Table:**

Bruker SMART APEXII CCD diffractometer	3561 independent reflections
Radiation source: rotating anode	2232 reflections with *I* > 2σ(*I*)
graphite multilayer	*R*_int_ = 0.052
Detector resolution: 10.0 pixels mm^-1^	θ_max_ = 28.4°, θ_min_ = 2.1°
φ and ω scans	*h* = −18→25
Absorption correction: multi-scan (*SADABS*; Bruker, 2009)	*k* = −11→11
*T*_min_ = 0.414, *T*_max_ = 0.899	*l* = −23→17
14696 measured reflections	

### Refinement

**Table:**

Refinement on *F*^2^	Primary atom site location: structure-invariant direct methods
Least-squares matrix: full	Secondary atom site location: difference Fourier map
*R*[*F*^2^ > 2σ(*F*^2^)] = 0.043	Hydrogen site location: difference Fourier map
*wR*(*F*^2^) = 0.094	H-atom parameters constrained
*S* = 1.01	*w* = 1/[σ^2^(*F*_o_^2^) + (0.0345*P*)^2^ + 1.0227*P*] where *P* = (*F*_o_^2^ + 2*F*_c_^2^)/3
3561 reflections	(Δ/σ)_max_ = 0.001
174 parameters	Δρ_max_ = 0.43 e Å^−^^3^
0 restraints	Δρ_min_ = −0.51 e Å^−^^3^

### Special details

**Table:**

Geometry. All esds (except the esd in the dihedral angle between two l.s. planes) are estimated using the full covariance matrix. The cell esds are taken into account individually in the estimation of esds in distances, angles and torsion angles; correlations between esds in cell parameters are only used when they are defined by crystal symmetry. An approximate (isotropic) treatment of cell esds is used for estimating esds involving l.s. planes.
Refinement. Refinement of F^2^ against ALL reflections. The weighted R-factor wR and goodness of fit S are based on F^2^, conventional R-factors R are based on F, with F set to zero for negative F^2^. The threshold expression of F^2^ > 2sigma(F^2^) is used only for calculating R-factors(gt) etc. and is not relevant to the choice of reflections for refinement. R-factors based on F^2^ are statistically about twice as large as those based on F, and R- factors based on ALL data will be even larger.

### Fractional atomic coordinates and isotropic or equivalent isotropic displacement parameters (Å^2^)

**Table:**

	*x*	*y*	*z*	*U*_iso_*/*U*_eq_	
Br1	0.746477 (15)	0.35697 (4)	0.531719 (18)	0.04220 (13)	
S1	0.58692 (4)	0.79730 (10)	0.30668 (4)	0.0302 (2)	
O1	0.49506 (9)	0.7539 (2)	0.50628 (9)	0.0242 (4)	
O2	0.61660 (12)	0.6477 (3)	0.27475 (11)	0.0467 (6)	
C1	0.55893 (14)	0.7536 (3)	0.39992 (14)	0.0224 (6)	
C2	0.59386 (14)	0.6560 (3)	0.45635 (14)	0.0210 (6)	
C3	0.65304 (14)	0.5643 (3)	0.45788 (14)	0.0233 (6)	
H3	0.6819	0.5542	0.4146	0.028*	
C4	0.66740 (13)	0.4885 (3)	0.52599 (15)	0.0249 (6)	
C5	0.62665 (13)	0.5023 (3)	0.59087 (15)	0.0236 (6)	
H5	0.6400	0.4501	0.6366	0.028*	
C6	0.56704 (14)	0.5911 (3)	0.58952 (15)	0.0215 (6)	
C7	0.55264 (13)	0.6627 (3)	0.52041 (14)	0.0195 (6)	
C8	0.50046 (14)	0.8064 (3)	0.43205 (15)	0.0239 (6)	
C9	0.52221 (14)	0.6084 (3)	0.65801 (15)	0.0286 (7)	
H9A	0.5216	0.7208	0.6742	0.043*	
H9B	0.5400	0.5417	0.6995	0.043*	
H9C	0.4757	0.5742	0.6452	0.043*	
C10	0.44272 (14)	0.9041 (4)	0.40406 (17)	0.0332 (7)	
H10A	0.4490	0.9265	0.3496	0.050*	
H10B	0.4410	1.0053	0.4324	0.050*	
H10C	0.3999	0.8455	0.4116	0.050*	
C11	0.65806 (14)	0.9260 (3)	0.33175 (15)	0.0258 (7)	
H11	0.6900	0.8687	0.3669	0.031*	
C12	0.63212 (15)	1.0808 (4)	0.36924 (17)	0.0356 (8)	
H12A	0.5815	1.0849	0.3686	0.043*	
H12B	0.6480	1.0883	0.4227	0.043*	
C13	0.66218 (16)	1.2156 (4)	0.32140 (17)	0.0371 (8)	
H13A	0.6312	1.3092	0.3205	0.044*	
H13B	0.7071	1.2495	0.3417	0.044*	
C14	0.66965 (16)	1.1439 (4)	0.24250 (18)	0.0385 (8)	
H14A	0.6252	1.1405	0.2156	0.046*	
H14B	0.7027	1.2055	0.2114	0.046*	
C15	0.69583 (15)	0.9771 (3)	0.25871 (15)	0.0298 (7)	
H15A	0.7459	0.9779	0.2669	0.036*	
H15B	0.6851	0.9037	0.2160	0.036*	

### Atomic displacement parameters (Å^2^)

**Table:**

	*U*^11^	*U*^22^	*U*^33^	*U*^12^	*U*^13^	*U*^23^
Br1	0.0393 (2)	0.0479 (2)	0.0394 (2)	0.01898 (17)	0.00616 (15)	0.00728 (15)
S1	0.0354 (5)	0.0370 (4)	0.0182 (4)	−0.0105 (4)	−0.0025 (3)	0.0023 (3)
O1	0.0247 (11)	0.0265 (11)	0.0214 (10)	−0.0013 (10)	0.0002 (8)	0.0014 (8)
O2	0.0728 (17)	0.0374 (13)	0.0298 (12)	−0.0144 (12)	0.0162 (11)	−0.0126 (10)
C1	0.0270 (16)	0.0230 (15)	0.0173 (14)	−0.0050 (13)	−0.0028 (12)	0.0002 (11)
C2	0.0232 (15)	0.0227 (15)	0.0169 (14)	−0.0049 (13)	−0.0009 (12)	−0.0021 (11)
C3	0.0249 (16)	0.0252 (15)	0.0199 (15)	−0.0049 (13)	0.0051 (12)	−0.0032 (12)
C4	0.0235 (16)	0.0217 (15)	0.0296 (16)	0.0029 (13)	−0.0017 (13)	−0.0022 (12)
C5	0.0305 (16)	0.0215 (14)	0.0189 (14)	−0.0019 (13)	0.0009 (12)	0.0010 (12)
C6	0.0267 (16)	0.0178 (14)	0.0199 (14)	−0.0047 (12)	0.0018 (12)	−0.0041 (11)
C7	0.0190 (15)	0.0193 (14)	0.0202 (14)	−0.0022 (12)	−0.0019 (12)	−0.0014 (11)
C8	0.0270 (17)	0.0226 (15)	0.0219 (14)	−0.0063 (13)	−0.0070 (12)	0.0015 (12)
C9	0.0303 (17)	0.0327 (17)	0.0229 (15)	0.0004 (14)	0.0052 (13)	0.0030 (12)
C10	0.0351 (18)	0.0300 (17)	0.0345 (18)	−0.0006 (14)	−0.0069 (14)	0.0026 (14)
C11	0.0230 (16)	0.0281 (16)	0.0262 (15)	0.0006 (13)	0.0001 (13)	0.0025 (12)
C12	0.0340 (19)	0.0309 (18)	0.0419 (19)	−0.0032 (15)	0.0092 (15)	−0.0070 (14)
C13	0.0345 (19)	0.0282 (18)	0.049 (2)	−0.0037 (15)	0.0022 (15)	−0.0062 (15)
C14	0.040 (2)	0.0314 (18)	0.044 (2)	−0.0028 (15)	−0.0053 (15)	0.0088 (15)
C15	0.0290 (17)	0.0310 (17)	0.0295 (16)	−0.0032 (15)	0.0038 (13)	0.0040 (13)

### Geometric parameters (Å, °)

**Table:**

Br1—C4	1.900 (3)	C9—H9B	0.9800
S1—O2	1.487 (2)	C9—H9C	0.9800
S1—C1	1.762 (3)	C10—H10A	0.9800
S1—C11	1.813 (3)	C10—H10B	0.9800
O1—C8	1.378 (3)	C10—H10C	0.9800
O1—C7	1.382 (3)	C11—C12	1.536 (4)
C1—C8	1.349 (4)	C11—C15	1.539 (4)
C1—C2	1.453 (4)	C11—H11	1.0000
C2—C7	1.384 (3)	C12—C13	1.521 (4)
C2—C3	1.388 (4)	C12—H12A	0.9900
C3—C4	1.380 (3)	C12—H12B	0.9900
C3—H3	0.9500	C13—C14	1.514 (4)
C4—C5	1.394 (3)	C13—H13A	0.9900
C5—C6	1.382 (4)	C13—H13B	0.9900
C5—H5	0.9500	C14—C15	1.512 (4)
C6—C7	1.380 (3)	C14—H14A	0.9900
C6—C9	1.494 (4)	C14—H14B	0.9900
C8—C10	1.477 (4)	C15—H15A	0.9900
C9—H9A	0.9800	C15—H15B	0.9900
			
O2—S1—C1	107.27 (13)	C8—C10—H10B	109.5
O2—S1—C11	106.84 (13)	H10A—C10—H10B	109.5
C1—S1—C11	97.83 (12)	C8—C10—H10C	109.5
C8—O1—C7	106.4 (2)	H10A—C10—H10C	109.5
C8—C1—C2	107.3 (2)	H10B—C10—H10C	109.5
C8—C1—S1	125.7 (2)	C12—C11—C15	106.4 (2)
C2—C1—S1	127.0 (2)	C12—C11—S1	110.44 (19)
C7—C2—C3	119.5 (2)	C15—C11—S1	109.33 (19)
C7—C2—C1	104.8 (2)	C12—C11—H11	110.2
C3—C2—C1	135.6 (2)	C15—C11—H11	110.2
C4—C3—C2	116.0 (2)	S1—C11—H11	110.2
C4—C3—H3	122.0	C13—C12—C11	105.0 (2)
C2—C3—H3	122.0	C13—C12—H12A	110.8
C3—C4—C5	123.5 (3)	C11—C12—H12A	110.8
C3—C4—Br1	118.4 (2)	C13—C12—H12B	110.8
C5—C4—Br1	118.1 (2)	C11—C12—H12B	110.8
C6—C5—C4	120.9 (2)	H12A—C12—H12B	108.8
C6—C5—H5	119.6	C14—C13—C12	104.4 (2)
C4—C5—H5	119.6	C14—C13—H13A	110.9
C7—C6—C5	114.8 (2)	C12—C13—H13A	110.9
C7—C6—C9	122.9 (2)	C14—C13—H13B	110.9
C5—C6—C9	122.3 (2)	C12—C13—H13B	110.9
C6—C7—O1	124.2 (2)	H13A—C13—H13B	108.9
C6—C7—C2	125.2 (3)	C15—C14—C13	103.0 (2)
O1—C7—C2	110.6 (2)	C15—C14—H14A	111.2
C1—C8—O1	110.8 (2)	C13—C14—H14A	111.2
C1—C8—C10	133.6 (3)	C15—C14—H14B	111.2
O1—C8—C10	115.5 (2)	C13—C14—H14B	111.2
C6—C9—H9A	109.5	H14A—C14—H14B	109.1
C6—C9—H9B	109.5	C14—C15—C11	104.4 (2)
H9A—C9—H9B	109.5	C14—C15—H15A	110.9
C6—C9—H9C	109.5	C11—C15—H15A	110.9
H9A—C9—H9C	109.5	C14—C15—H15B	110.9
H9B—C9—H9C	109.5	C11—C15—H15B	110.9
C8—C10—H10A	109.5	H15A—C15—H15B	108.9
			
O2—S1—C1—C8	−139.5 (2)	C3—C2—C7—C6	3.7 (4)
C11—S1—C1—C8	110.1 (3)	C1—C2—C7—C6	−177.7 (3)
O2—S1—C1—C2	40.8 (3)	C3—C2—C7—O1	−177.3 (2)
C11—S1—C1—C2	−69.6 (3)	C1—C2—C7—O1	1.3 (3)
C8—C1—C2—C7	−1.7 (3)	C2—C1—C8—O1	1.5 (3)
S1—C1—C2—C7	178.1 (2)	S1—C1—C8—O1	−178.30 (18)
C8—C1—C2—C3	176.6 (3)	C2—C1—C8—C10	−177.6 (3)
S1—C1—C2—C3	−3.6 (5)	S1—C1—C8—C10	2.6 (5)
C7—C2—C3—C4	−1.9 (4)	C7—O1—C8—C1	−0.7 (3)
C1—C2—C3—C4	−179.9 (3)	C7—O1—C8—C10	178.6 (2)
C2—C3—C4—C5	−0.9 (4)	O2—S1—C11—C12	−177.07 (19)
C2—C3—C4—Br1	178.6 (2)	C1—S1—C11—C12	−66.3 (2)
C3—C4—C5—C6	2.1 (4)	O2—S1—C11—C15	66.2 (2)
Br1—C4—C5—C6	−177.4 (2)	C1—S1—C11—C15	177.0 (2)
C4—C5—C6—C7	−0.4 (4)	C15—C11—C12—C13	−5.9 (3)
C4—C5—C6—C9	−180.0 (2)	S1—C11—C12—C13	−124.4 (2)
C5—C6—C7—O1	178.7 (2)	C11—C12—C13—C14	28.9 (3)
C9—C6—C7—O1	−1.7 (4)	C12—C13—C14—C15	−41.2 (3)
C5—C6—C7—C2	−2.5 (4)	C13—C14—C15—C11	37.0 (3)
C9—C6—C7—C2	177.1 (3)	C12—C11—C15—C14	−19.3 (3)
C8—O1—C7—C6	178.5 (2)	S1—C11—C15—C14	100.0 (2)
C8—O1—C7—C2	−0.4 (3)		

### Hydrogen-bond geometry (Å, °)

**Table:**

*D*—H···*A*	*D*—H	H···*A*	*D*···*A*	*D*—H···*A*
C5—H5···O2^i^	0.95	2.60	3.465 (3)	152.
C9—H9B···O2^i^	0.98	2.55	3.489 (3)	161.
C10—H10B···O1^ii^	0.98	2.60	3.480 (3)	149.

Symmetry codes: (i) *x*, −*y*+1, *z*+1/2; (ii) −*x*+1, −*y*+2, −*z*+1.
